# Drinking motives and alcohol intervention for patients with HIV

**DOI:** 10.1186/1940-0640-10-S2-P6

**Published:** 2015-09-24

**Authors:** Jennifer C Elliott, Efrat Aharonovich, Ann O'Leary, Deborah S Hasin

**Affiliations:** 1 Department of Psychiatry, Columbia University Medical Center, New York, NY, USA; 2 Division of Clinical Phenomenology, New York State Psychiatric Institute, New York, NY, USA; 3 Centers for Disease Control and Prevention, Atlanta, GA, USA; 4 Department of Epidemiology, Mailman School of Public Health, Columbia University, New York, NY, USA

## Background

For individuals with HIV, heavy drinking can interfere with medication adherence and impair liver function. Yet, many individuals with HIV drink heavily. A recent alcohol intervention trial[1] indicated that motivational interviewing (MI) enhanced with HealthCall (consisting of self-monitoring and discussion of drinking data collected through self-monitoring) was effective at reducing drinking in HIV patients. Also using this data, Elliott et al.[2,3] showed that patients' drinking motives at baseline were associated with both past-year and end-of-treatment drinking. However, it remains unknown: (a) whether motivational interventions also decreased drinking motives, and (b) whether the predictive validity of motives extended to end-of-study (i.e., 12-months post-baseline).

## Materials and methods

The sample consisted of 254 HIV-infected patients with past-month heavy drinking (78% male; 94.5% minority), participating in a randomized trial of brief alcohol interventions[1]. Participants completed one of three conditions: (a) a DVD educational control; (b) MI only; (c) MI+HealthCall. Patients reported motives, drinking, and alcohol dependence symptoms at baseline, end-of-treatment, and end-of-study.

## Results

The intervention conditions evidenced few differences in motives at end-of-treatment (MI+HealthCall evidenced higher drinking due to social pressure, p<0.05), and no differences at end-of-study. However, baseline motives remained predictive of drinking at end-of-study (drinking to cope with negative affect associated with more past-month drinks and dependence symptoms, ps<0.05; drinking due to social pressure with fewer drinks, p<0.01).

## Conclusions

Although MI+HealthCall reduces drinking, it does not reduce drinking motives. Individuals participating in MI+HealthCall were more likely to transition to drinking due to social pressure, an indicator of lower-risk drinking[2]. However, motives (particularly drinking to cope) were predictive of alcohol consumption and dependence up to a year later, suggesting their importance in understanding and predicting drinking. Further work should increase attention to drinking motives in alcohol interventions for HIV patients.

## Trial registration

Clinicaltrials.gov: NCT00371969.
